# New evidence for regional pastoral practice and social complexity in the Eastern Tianshan Mountains in the first millennium BCE

**DOI:** 10.1038/s41598-023-31489-9

**Published:** 2023-03-16

**Authors:** Yuxuan Wang, Francesca Monteith, Tongyuan Xi, Meng Ren, Daren Li, Songmei Hu, Jianxin Wang, Marcella Festa, Jian Ma

**Affiliations:** 1 China-Central Asia “the Belt and Road” Joint Laboratory on Human and Environment Research, Northwest University, Xi’an, China; 2 Key Laboratory of Cultural Heritage Research and Conservation, School of Culture Heritage, Northwest University, Xi’an, China; 3 Shaanxi Academy of Archaeology, Xi’an, China

**Keywords:** Ecology, Zoology

## Abstract

Mobile pastoralism was a key lifeway in the Late Bronze and Iron Age of Northwest China and played a crucial role in the regional socio-cultural development, as well as the formation of transregional networks. In this paper we analyse the complete faunal assemblage from House F2 in Shirenzigou, on the Eastern Tianshan Mountains, in combination with radiocarbon dating and spatial analysis, to explore local animal resources exploitation strategies and related socio-economic implications. Our results show an intensive multipurpose caprine management, while the exploitation of other domestic taxa, cattle, horses and dogs, was limited. This pastoral economy was supplemented with some hunting. The differentiated use of space in F2 indicates that basic domestic tasks were carried out in the structure, however its position within the landscape and the predominance of bone tools related to warfare and socialization activities, suggests that it was not an ordinary dwelling, it may also have served as a watch post for the summer encampment within the gully. Our findings constitute an important contribution on the discussion on animal resources exploitation strategies and their relationship with evolving socio-economic complexity in the Eastern Tianshan region in the late first millennium BCE.

## Introduction

Mobile pastoralism played a crucial role in the formation of cultural and trade networks across the Eastern Steppes in the first millennium BCE^1–3^. Mobile pastoralists’ lifestyle fostered the movements of people, goods, domestic animals and plants, bringing about profound changes in the subsistence strategies, craft production, and social organization across this region^4–6^. Archaeological research in the Tianshan Mountains of Xinjiang, in Nortwest China, has demonstrated that this region and its pastoral peoples were main players in these exchanges^7–11^. Understanding these early pastoral societies and their economy is, therefore, particularly important for the study of late Eurasian prehistory.

Zooarchaeological research in Xinjiang has provided a window into the subsistence strategies and herd management of pastoralist and agro-pastoralist groups, who appear to have relied on the husbandry of a suite of animals, including cattle, horses, sheep, goats and dogs^12,13^. A growing corpus of isotopes and dental pathology analyses has enhanced our knowledge of domestication and ancient diets^14–17^. These methods have shown that by the first millennium BCE domesticated bovids were ubiquitously exploited to varying degrees of intensity in Northwest China. Horses were also domesticated and ridden at this time^18^.

Zooarchaeological research in this region is, however, fragmentary and largely conducted on small and/or selected faunal assemblages that do not reflect the full animal representation at the sites^12,18,19^. There are significant lacunae, especially concerning mortality profiles for late prehistory, which impedes an adequate understanding of animal use both within and between sites. Issues with past recovery, archival, and analysis protocols in Central Asia and Northwest China have previously been highlighted^20,21^. Research focus has also been on long-distance interactions and relatively large-scale variations in pastoralists’ strategies^1,2,22,23^. The dearth of micro-scale zooarchaeological research means there are few studies of localized animal use, and little examination of the relationship between subsistence economies and local systems of social organization. Research into bone working in China during the Late Bronze and Iron Ages are primarily focused on the Yellow River valley and the Wei River valley^24–27^. Moreover, worked bones are generally evaluated in isolation from both unworked bones, other artifacts and their contexts within sites^28^, which significantly limits research into the range of faunal exploitation strategies and prevents a substantial understanding of localized past behaviors and social relationships^29,30^.

**Figure 1:**
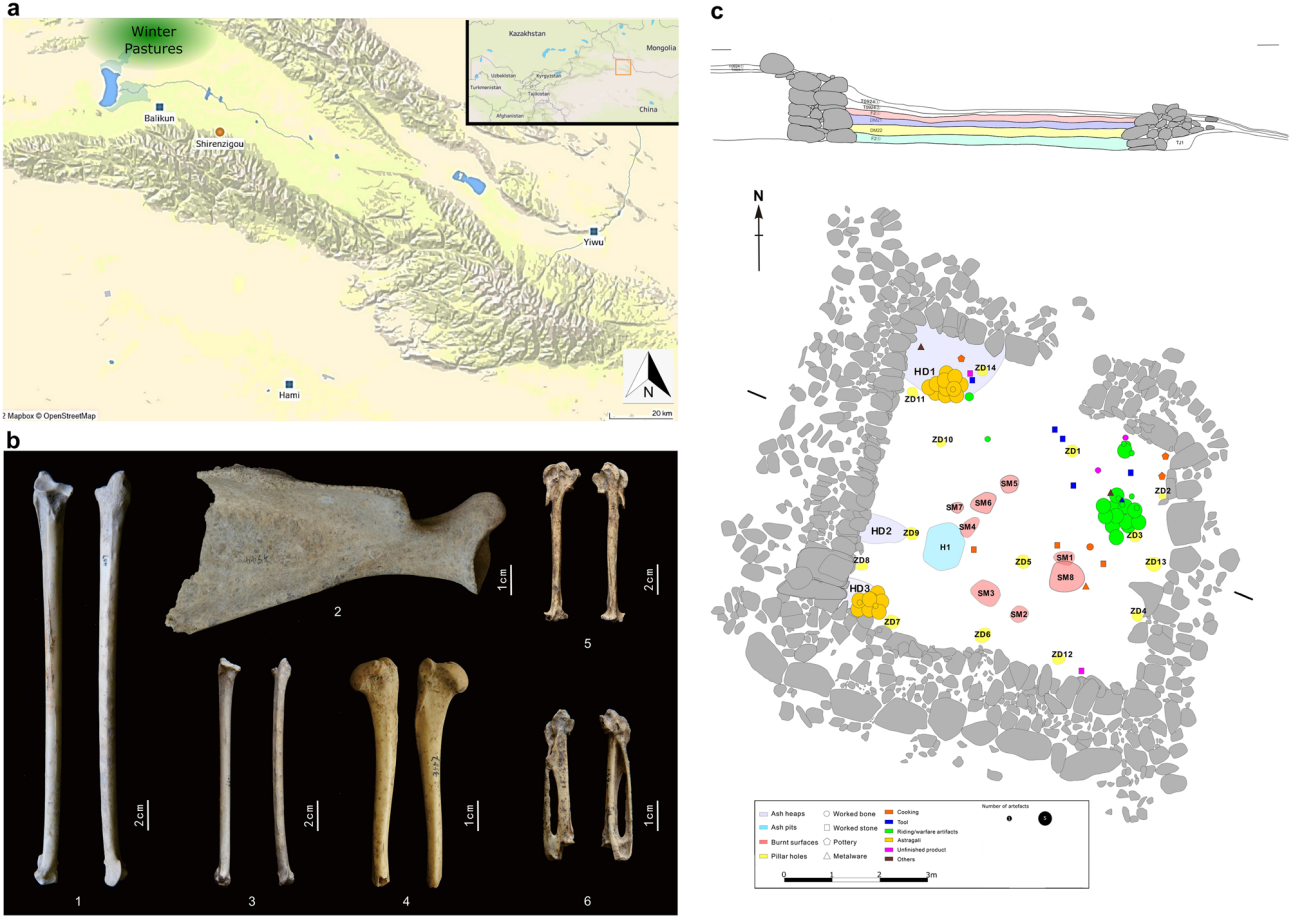
(**a**) Location of Shirenzigou. The map was created by F.M. using Tableau 2014.4 https://www.tableau.com/2021-4-features. (**b**) Bones from F2: (1) Ulna, Golden eagle (*Aquila chrysaetos*); (2) Scapula, Sheep (*Ovis aries*); (3) Ulna, Black kite (*Milvus migrans*); (4) Humerus, Dog (*Canis familiaris*); (5) Carpus, Golden eagle (*Aquila chrysaetos*) (6) Carpus, Large-billed crow (*Corvus macrorhynchos*) (Photo credit: Y.W. and J.M). (**c**) Profile and plan of F2 showing artefacts and features from across all layers .The plan was mapped by J. M. and Y. W. and digitalized using CorealDraw X8 https://www.coreldraw.com/en/pages/coreldraw-x8/.

This study presents and discusses the complete faunal assemblage from a structure (hereafter referred to as F2) from the Shirenzigou site, in the northern foothills of the Eastern Tianshan Mountains^31^ (Fig. 1a). Faunal evidence was examined and subsequently contextualized chronologically, through calibrated radiocarbon dating, and spatially, through interviews with the excavators, in order to explore the local subsistence strategies and related socio-economic implications. This study provides a nuanced insight into the relationship between animal use and the complexity of transhumanant societies in the Eastern Tianshan in the first millennium BCE.

### Shirenzigou

The Shirenzigou site (also referred to as Dongheigou) is located in the northern foothills of the Tianshan Mountains, in present day Balikun County. The landscape around the site consists of mountain meadows and gravel glacial outwashes. To the north, there is a broad valley into which alluvial flans spread. Although the lower fluvial plain lies at a height of 1800–2000 m and hosts irrigated wheat cultivation in modern times, there is no indication for historic irrigation. The mountains to the north, which rise to a height of over 3600 m are forested with permafrost on their peaks. The steady water supply and good drainage make this area an ideal summer pasture. The population appears to be transhumant, collating in the northwest extremity of the lowlands during the winter and spreading into the foothills during the summer. Such practices are well recorded in other locales^32^ (Fig. 1a).

From 2005 onwards surveys and excavations conducted by Northwest University and other institutions in Shirenzi Village uncovered a settlement with an area about 8.75km^2^ in the Shirenzigou locales. Archaeological evidence has suggested that the site was a pastoral settlement with an associated cemetery used seasonally for several centuries. Carbon dates for the site place its occupation at ca. 1300 BCE–300 CE, which covers the local Bronze and Iron Ages. A cluster of six structures (F2–F7) was attributed to the latter phase (400 BCE–300 CE)^10,31,33–36^. The zooarchaelogial analysis conducted to date has been on a whole site-basis and indicates intensive use of caprine over time for subsistence and tool making^12,28,34,37,38^.

The faunal remains examined in this paper come from the excavation of a single structure, F2, in 2009^31^ (Fig. 1b). F2 is a nearly-square semi-subterranean single-room structure with its main door in the north wall. During excavation, human activities were identified in the second, third and fourth layers (Fig. 1c). The earliest layer consists of fireplaces (SM5-8) and an ash heap (HD3) and does not appear to have been an occupation, but part of the construction process. DM22 represents the first occupation of F2 and includes pillar holes (ZD1–ZD14), fireplaces (SM3–SM4) and an ash pit (H1); The secondary occupation (DM21) includes burnt surfaces (SM1–SM2) and ash heaps (HD1–HD2), some of the post holes from DM22 may also relate to this phase of occupation. F2 is located in a high position, at the edge of a slope on the eastern side of a seasonal stream. While usually the houses in Shirenzigou are open to the east^36^, the northward door of F2 would have provided no shelter from the prevailing northerly winds. For these reasons, the excavators suggest that F2 may have served as a lookout^31^.

## Results

### Zooarchaeological analysis

The faunal assemblage contained a total of 1032 fragments, 187 were identified to species and 253 were worked bones. There was a fairly high degree of fragmentation, with 62.6% of the bones being less than 0.25 of the total bone (or shorter than 5 cm). The rate of weathering (1.2% of fragments) and gnawing (1.9% of fragments) were low. Butchery marks were also scarce, present on only 1.3% of fragments. They were all shallow cut and scrape marks, but there was no clear pattern to these marks in terms of taxonomic and skeletal distribution. No traces of charring were identified (Table S1). The non-worked faunal assemblage was dominated by mammals (97.9%NISP), although at least 3 species of birds were identified, *Aquila chrysaetos* (1.1%NISP), *Milvus migrans* (0.5%NISP) and *Corvus macrorhynchos* (0.5%NISP). Caprines were prevalent (86.3%NISP), followed by cattle (8%NISP). Other domestic species included dog (1.6%NISP) and horse (1%NISP). Wild taxa mostly consisted of cervids and gazella and were less represented (< 1%NISP) (Table 1).

**Table 1:** NISP and MNI for the assemblage in F2, Shirenzigou.

Category	Taxon	NISP	NISP %	MNI	MNI%
Domestic species		181	96.9	32	86.5
	Caprine, *Ovis aries*/*Capra hircus*	161	86.3	29	78.4
	Cattle, *Bos taurus*	15	8	1	2.7
	Dog, *Canis familiaris*	3	1.6	1	2.7
	Horse, *Equus caballus*	2	1	1	2.7
Wild Species		6	3.1	5	13.5
	Deer, *Cervidae* sp.	1	0.5	1	2.7
	Gazelle, Gazella genus	1	0.5	1	2.7
	Golden eagle, *Aquila chrysaetos*	2	1.1	1	2.7
	Black kite, *Milvus migrans*	1	0.5	1	2.7
	Large-billed crow, *Corvus macrorhynchos*	1	0.5	1	2.7
Grand Total		187	100	37	100

MNI of domesticated animals (cattle, caprine, dog, and horse) was generally consistent with the NISP, with caprine being the best represented category (78.4%MNI). However, cattle were less represented by MNI (n = 1; 2.7%MNI). MNI for cattle was given by 15 different skeletal elements all from the right side and, judging by quality, size and fusion rate, could have belonged to a single individual. Other faunal categories were evenly represented at 2.7%MNI (Table 1).

**Figure 2:**
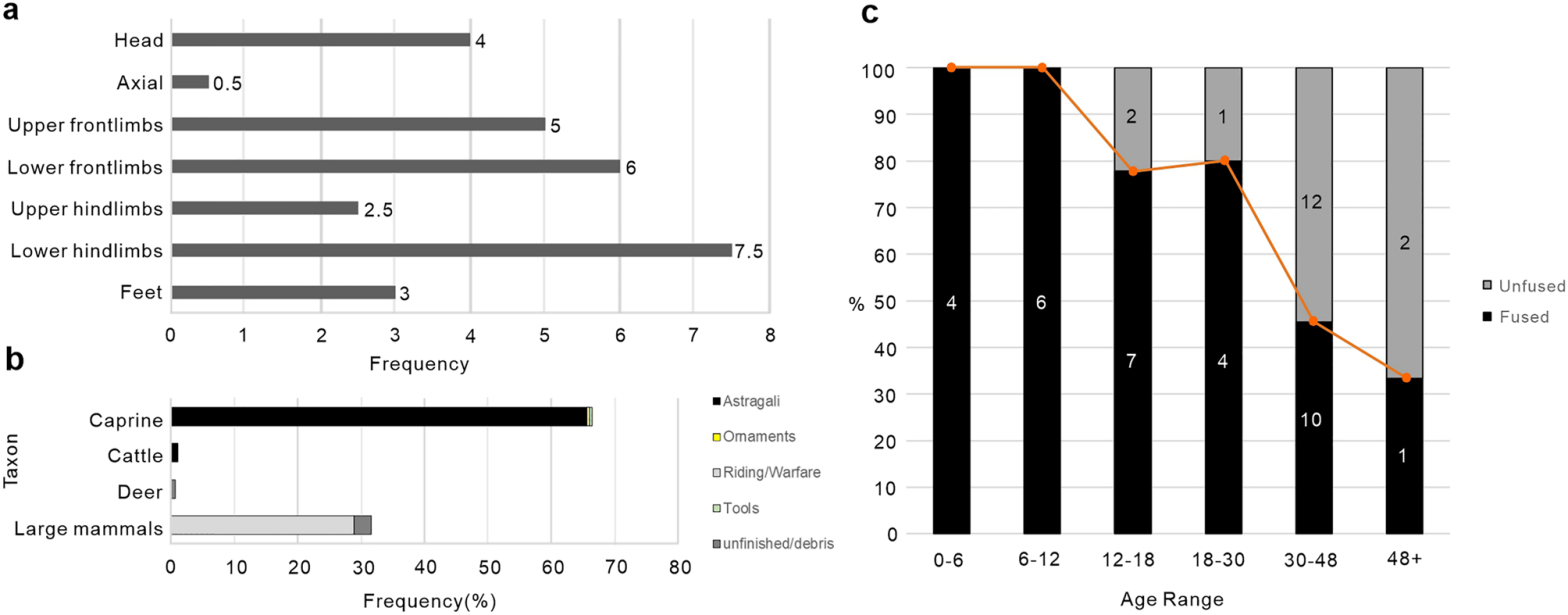
(**a**) Anatomical region distribution for caprine, adapted from^81^. (**b**) Frequency (%) of different types of worked bones per taxa. For data see Tables S2 and S3. (**c**) Caprine survivorship from epiphyseal fusion, using^82^. Age class 0–6 (A) was defined by the fusion staus of the proximal radius (n = 4); Age class 6–12 (B) was defined by the fusion staus of the distal humerus (n = 4) and scapula (n = 2); Age class 12–18 (C) was defined by the fusion staus of the first (n = 4) and second (n = 5) phalanges; Age class 18–30 (D) was defined by the fusion staus of the distal metatarsal (n = 2) and tibia (n = 3); Age class 30–48 (E) was defined by the fusion staus of the distal femur (n = 3), radius (n = 5) and proximal femur (n = 6), tibia (n = 3) and ulna (n = 3) and the calcaneus (n = 2); Age class 48 + (F) was defined by the fusion staus of the proximal humerus(n = 3). Numbers in the stacks indicate the raw data, while the red line represents the survivorship curve per percentage.

MNE for caprine showed a fair representation of the whole body, except for the axial region, which accounted for a significantly lower BPR (0.5). Limbs were well represented, including meaty parts and body sections usually used to make tools, such as the knee bones and metapodials. The upper hindlimbs were comparatively less represented, plausibly because this body region only includes the femur. Feet and head were also present in the assemblage (Fig. 2a).

Caprine mortality profile, generated on the basis of epiphysial fusion of 49 bones, showed a relatively late killing pattern. The majority died aged to 30–48 months, with a quarter of the sample surviving past 48 months. A slight “uptick” between groups 12–18 months and 18–30 months is probably due to the small sample size (Fig. 2c). Our results may have also been affected by preservation biases against unfused bones, however, they are consistent with those of dental analysis conducte, yet, on a small sample (n = 8): all specimens survived past 24 months and two lived beyond 48 months.

### Worked bones

253 worked elements, including bone, horn and antler, were unevenly represented taxonomically, with a higher proportion of caprine bones (66.4% of the worked assemblage). Cattle accounted for 1.2% of the worked bones, while artifacts made out of skeletal elements from unidentified large mammals made up 31.2%. Caprine horns and deer antlers accounted for 0.4% and 0.8% of the worked specimens, respectively. Overall, the taxonomic proportion for worked bones mirrored the assemblage of non-worked bones (Fig. 2b).

**Figure 3:**
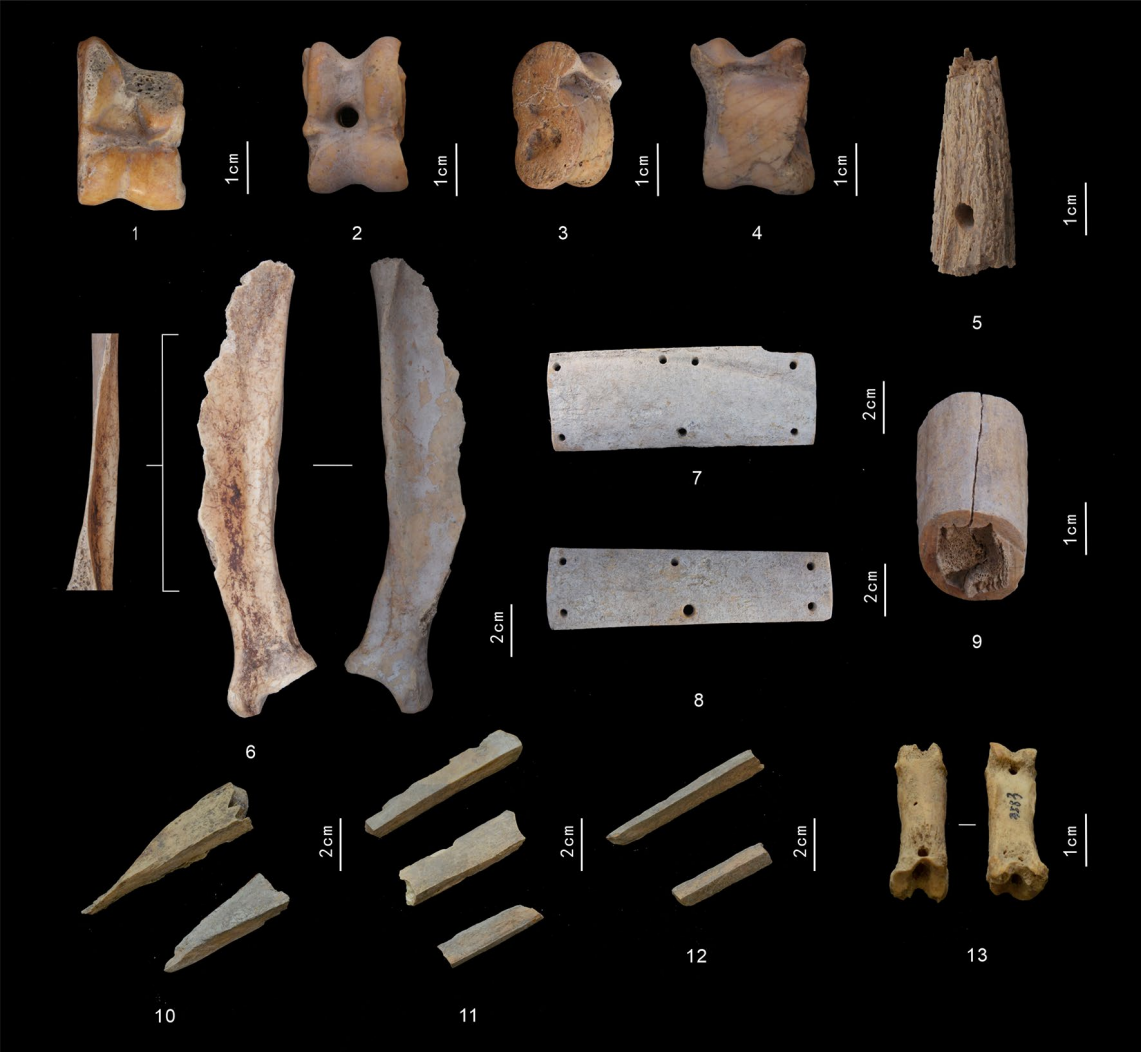
Worked bones in Shirenzigou F2: (**1–4**) Astragali; (**5**) Horn bit; (**6**) Bone knive; (**7, 8**) Plaques; (**9**) Antler unfinished product; (**10–12**) Bone unfinished products; (**13**) Ornament. Photograph credit: Y. W.

**Figure 4:**
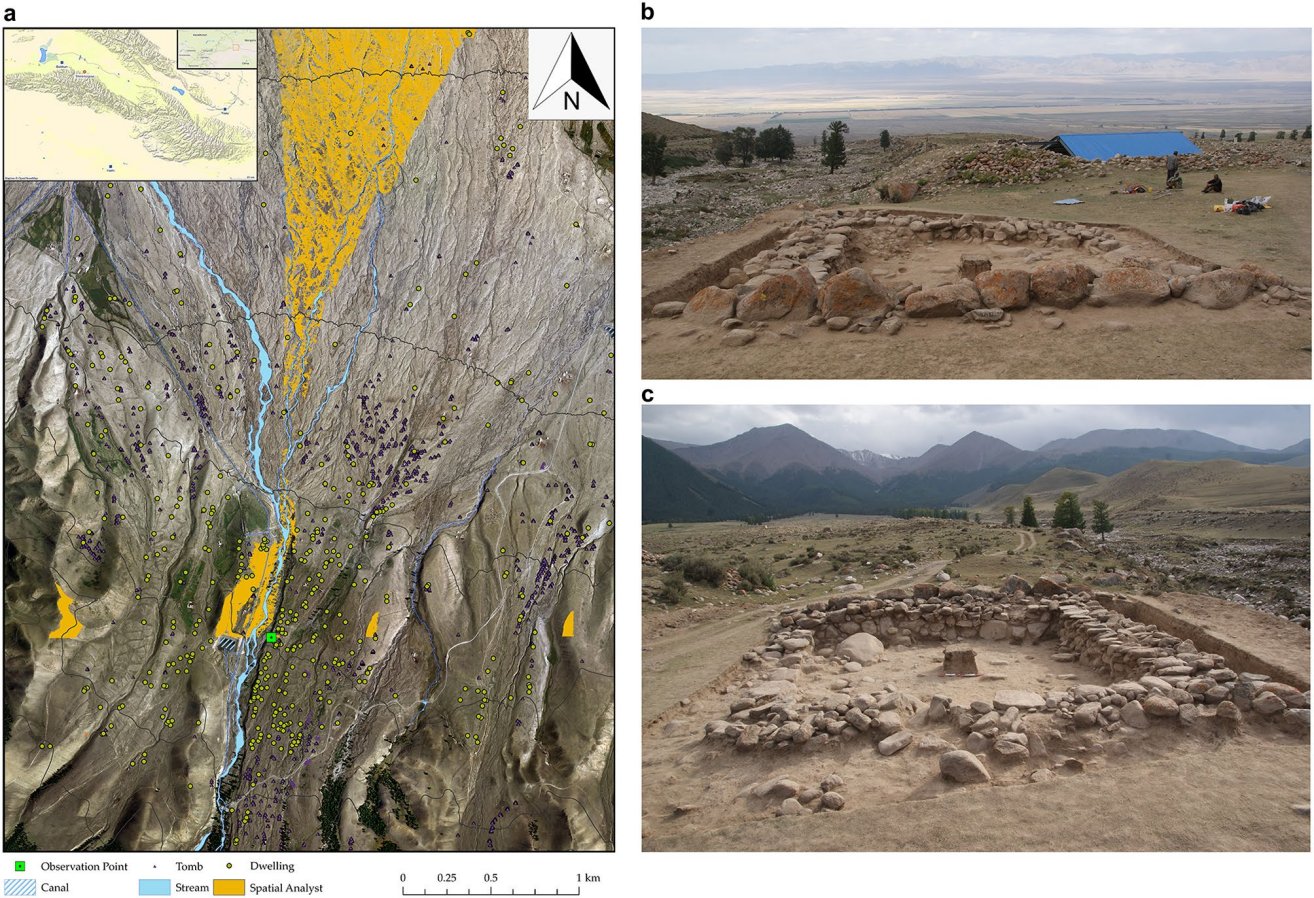
(**a**) Satellite map of the Shirenzigou site showing the location of the site. The other dwellings (yellow circles) and tombs (purple triangles) identified at the site are also marked. The viewshed from the north door of the dwelling is shown in orange. The map was created by D. L. and F. M. using ArcGIS 10.2 https://www.esri.com. The base map is the aeral photograph taken during our survey in 2010. (**b**) View from the south of the excavation looking north over the fluvial plain, note the river bed to the left. (**c**) View from the north of the dwelling looking south towards the mountains. Photograph credit: J. M.

The worked elements were divided into the following categories: “astragali”, “riding/warfare”, “tools”, “ornaments”, and “unfinished/debris”. “Astragali” are decorated animals knee bones still used today to play traditional games, such as the *Asyk*, in rural areas of Central Asia, or the Tibetian *dmag the*^39,40^. Astragali are used in both gambling and to solve disputes in and between villages^39,40^. They, therefore, have distinctive cultural, religious and social meaning. The discovery of astragali in funerary contexts, at the feet of the deceased or used as amulets in Xinjiang supports their socio-ritual significance^41–43^. 169 astragali made up 66.8% of the worked bone assemblage. The overwhelming majority were caprine bones (n = 166, 65.6%), with only 3 cattle specimens found in F2 (1.2%). Four working processes were identified: perforation, carving, cutting and polishing, the latter having being performed simultaneously to the other manufacturing techniques (Fig. 3—1–4).

**Figure 5:**
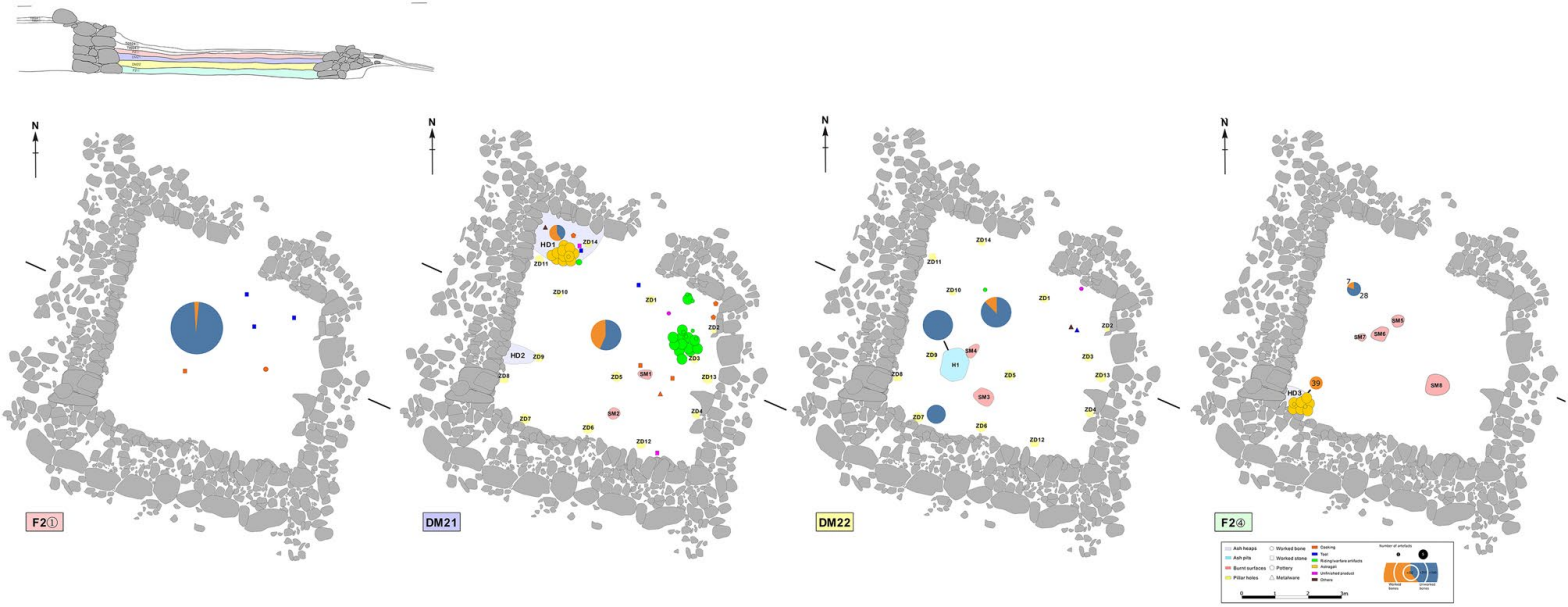
Plan of F2 divided according to stratigraphy with features and artefacts marked on each layer. The plan was mapped by J. M. and Y. W. and digitalized by J. M., Y. W. and F. M. using CorelDraw X8 https://www.coreldraw.com/en/pages/coreldraw-x8/.

**Figure 6:**
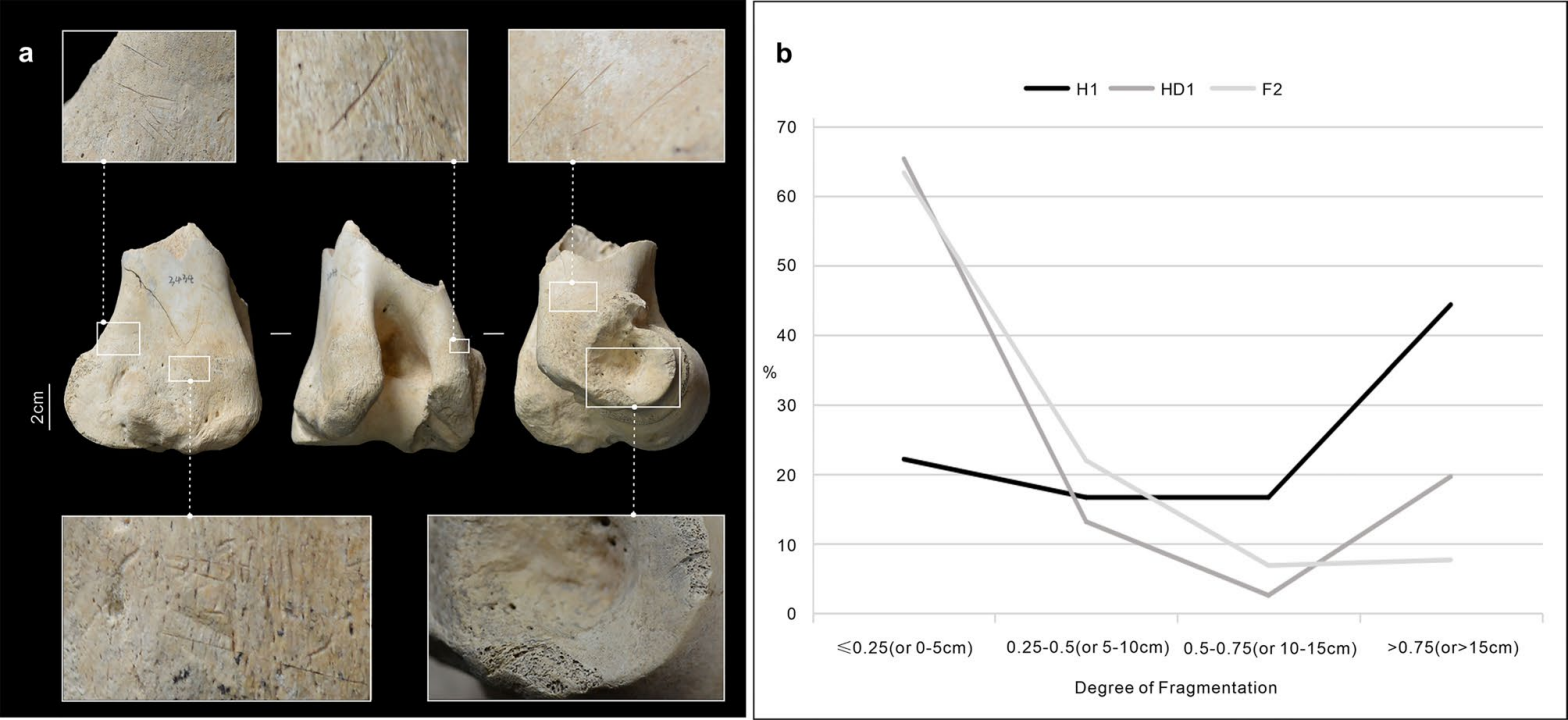
(**a**) Cut marks and their location on cattle humerus distal end. Photograph credit: Y. X. (**b**) Degree of fragmentation in different features (H1 and HD1) of F2 compared with faunal remains from all other contexts. Proportion of bone has been used for identified bones, while cm is used for unidentified bones.

**Figure 7:**
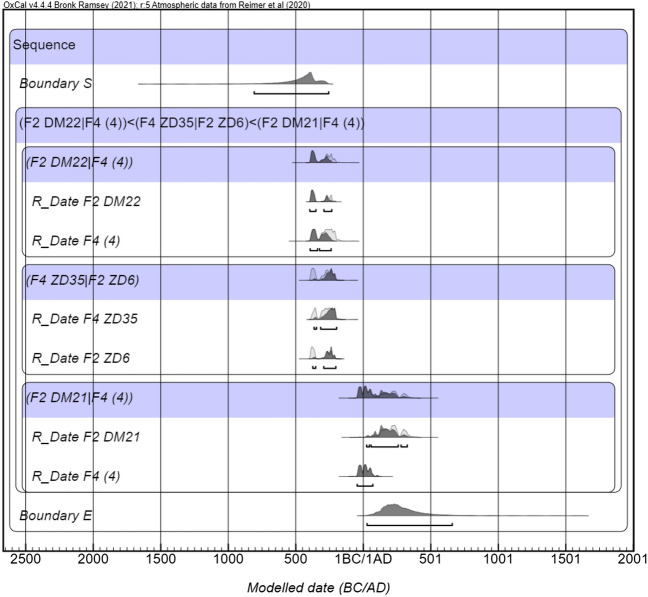
Calibrated radiocarbon dates for Shirenzigou for F2 and F4. Accelerator Mass Spectrometry Dating Laboratory of Peking University and calibrated using OxCal and IntCal20^51,52^.

“Riding/warfare” comprised plaques and one horn bit. 72 bone plaques made up 28.5% of the worked assemblage. The plaques were rectangular in shape and variously perforated in terms of location and number of holes. While the majority were plain, measuring between 10 and 13 cm in length, 7 longer specimens (ca. 18 cm in length) were curved. 59 plaques were found one next to the other, suggesting that they were tied together throught the perforations. Other smaller groups of plaques (n = 7; n = 3) had been arranged in a similar manner^31^. Similar artefacts dating to the first millennium BCE have been recovered from burial contexts in Northern China, Siberia and Central Asia, and identified as torso armors^28,44^. The plaques could not be identified taxonomically beyond the category of “large mammal”, however, cattle and deer could have been the sources, as they are fairly well-represented in the worked and non-worked bones assemblages (Fig. 3—7, 8). The horn bit, made of caprine horn, was a slightly conical cylinder trimmed at both ends with a horizontal perforation through the broader end. Similar objects made of various materials have been found in Early Iron Age sites across Northern China^45,46^ (Fig. 3—5).

The “tool” category consists of a single knife made with a sheep scapula. The anterior margin and the spine of the scapula had been removed, while the glenoid process and the posterior section of the blade had been polished and sharpened (Fig. 3—6).

One item included in the “ornaments” group was a double pierced caprine first phalanx. The two perforations, one longitudinal and one slightly oblique intersect with each other, allowing it to be suspended in multiple ways, which indicates it was a pendant or a toggle (Fig. 3—13).

7 bone and 2 antler fragments carrying toolmarks were classified as “unfinished/debris” (Fig. 3—9–12). Their discovery suggests a possible domestic production. The assemblage was too small to provide a fair representation of the bone production. However, these unfinished items are comparable in terms of shape, size and type and location of the toolmarks with those recovered in several Iron Age agro-pastoralist sites in the hilly region north of Zhouyuan (Northwestern Shaanxi)^27,47^. The stages of tool making have been reconstructed for these sites, the final products obtained being arrowheads. Although no finished arrowheads were recovered in F2, specimens that are consistent with our finds in terms of size and material have been found elsewhere in the site^28^. 9 horn fragments found associated to worked elements in DM21 lacked any toolmarks: they may have been collected with the intention of being worked.

### Spatial analysis

F2 is located on the primary river terrace of a relatively narrow valley, which runs south to north. This valley does not lead to a pass across the mountains and, although there are some as yet unexcavated tombs to the south of the site, there is no indication that F2 was intended to prevent persons from travelling further into the valley. Although its location is higher than the lower fluvial plain, it is in a low position within its own valley, meaning that its viewshed was restricted. The main door provides a clear view of the meadows on the western slope of the valley, which could have been used, and likely contested, by several different communities, as happens today^9,48^. However, the position of F2 to the east of the river means that it would have been difficult for anyone located there to police access to the pastures. The north door of F2 has a clear viewshed of the entrance to the valley and the flatland upon which a cluster of buildings have been excavated. It is therefore possible that this structure was intended to function as a lookout post for the gully, which appears to have formed a defensible location for a summer encampment (Fig. 4).

The contexts of the artefacts recovered during excavation were recorded to the nearest cm. Unworked faunal remains and ceramic sherds were recorded by number to the layer or feature in which they were recovered. The occurrence of faunal remains and ceramic sherds was inconsistent across the occupation phases (Fig. 5; Table S3).

76 broken animal bones and 109 worked astragali were unearthed in HD1, in the northwestern corner of F2. The broken elements mostly consisted of meaty sections of the animals’ body (75%), while phalanges and mandibles made up the 25% of the total. 91.1% of the elements recovered from HD1 were mammal limbs bones, ribs, vertebrae and mandibles, which were very fragmented for taphonomic reasons, and which would, therefore, be unsuitable for tools production (Fig. 6b). Elements, such as scapulae, ulnae, phalanxes and well-preserved mammalian limb bones, that could be potentially used to make tools only represented 7.9% of the total. In addition to animal bones, broken stone pestles, three broken plaques, copper slag and several pottery shards were unearthed in HD1. The overall picture would suggest that HD1 was a trash heap. HD1 also contained 109 worked astragali, which may have been discarded.

The ash deposit HD3, in the southwestern corner of F2, also included a concentration of 39 astragali, however, since no other artefacts were found on this layer, it is unclear if it is an occupation layer. The deposition of these astragali may represent a foundation ritual of some sort.

18 bones unearthed from H1: 12 have been identified as cattle right front and hindlimbs elements, and 6 as large mammals long bone shafts. The fragmentation rate of these bones was relatively low (Fig. 6b). 61.1% of the bones were of a suitable quality for making tools, however cut and scrape marks on the distal humerus, calcaneum and carpi are consistent with disarticulation and/or defleshing activities with small metal knives^49,50^ (Fig. 6a), similar to the one found on the floor in the southwestern quadrant of the center of DM21. This knife was closely associated with two fireplaces (SM1 and SM2). Similarly, there were two burnt surfaces (SM3, SM4) located to the northeast and southeast of H1, in the southeastern quadrant of the center of DM22, further suggesting that the center of the dwelling was devoted to food processing in both phases of occupation. This arrangement of hearths in the centre of the dwelling with refuse in a corner is replicated in several structures in Shirenzigou^36^.

In the northeastern corner of F2, 7 worked plaques and some unfinished products were discovered associated with several pottery vessels, one bronze handle, one iron awl and one stone wheel which are located across both occupation phases. Moving slightly to the south of the eastern wall, other 59 plaques were found, suggesting that this whole area was devoted to manufacture.

### Chronology

There are three radiocarbon dates for F2. These provide dates for 3 contexts, DM21, DM22 and ZD6. Theoretically these three dates have a clear stratigraphy. ZD6 is a post hole which passes through the surface DM22, but which does not impose upon the surface DM21. However, the radiocarbon dates for ZD6 (2270, 25) and DM22 (2270, 20) are almost identical. The calibration curve for this period is relatively flat, meaning that these radiocarbon dates may date to a period of over 180 years (c. 396–210 BCE). The date for ZD6 could be, therefore, that of the wood used to construct the structure, however if the wood was charred then the date from ZD6 would represent the *terminus ante quem* for the occupation represented by DM22.

The date for DM21 ((1830, 20) Cal.129–312 CE) is very late within the overall chronology of the site and while it is possible that it is a contaminated sample, it matches the date ranges of the radiocarbon dates for F4, which is located south-east of F2. There are three dates for F4, which date F4(4), a post hole associated with it (ZD35) and a sample from layer F4(3). As with F2 the radiocarbon dates for the occupation layer (2240,35) and the post hole (2230,25) are very similar. This indicates that the post hole represents a *terminus ante quem* for the context F4(4). The date taken from layer directly above the layer F4(4) is similar to that of DM21. This indicates that this structure would have been abandoned before the second phase of occupation of F2 (Table 2; Fig. 7). These dates demonstrate that these structures were used intermittently with intervals running to hundreds of years.

**Table 2:** Calibrated radiocarbon dates for Shirenzigou F2 and F4.

Laboratory Number	Context	Material	Radiocarbon dates (BP)	Calibrated Radiocarbon dates at 94.5% (BCE)
BA111923	F2 DM22	Animal bone	2270 ± 20	395-211 BCE
BA111924	F2 ZD6	Charcoal	2270 ± 25	396-209 BCE
BA111928	F2 DM21	Animal bone	1830 ± 45	129-312 CE
BA111935	F4(4)	Wood	2240 ± 35	375-211 BCE
BA111947	F4 ZD35	Wood	2230 ± 25	311-179 BCE
BA111934	F4 (4)	Animal Bone	2000 ± 50	46 BCE-73 CE

Accelerator Mass Spectrometry Dating Laboratory of Peking University and calibrated using OxCal and IntCal20^51,52^.

## Discussion

The faunal assemblage from F2 reflects a pastoral economy, based on the exploitation of domestic taxa, caprine, cattle, horse and dog. The relative faunal proportion is comparable to that observed in other Iron Age structures in Shirenzigou, such as F4[^37^; Fig. S1a], and in individual modern households of pastoralists that practice vertical seasonal transhumance^48^. Herding was supplemented by hunting, as suggested not only by remains of deer and other wild taxa in the assemblage, but also the presence of large and medium raptors, as falconry is well recorded in ancient and modern Northern China and Central Asia^53–55^.

Ethnoarchaeological research suggests that such birds were captured at a young age and kept for several years, before being released^54^. This might explain the scarcity of raptors bones in our assemblage. Biometrical data for golden eagles are consistent with those of adult specimens, which may have died in the site prematurely. While several metal plaques representing raptors were discovered in funerary contexts in Shirenzigou^36^, no distinctive tools related to falconry were found at the site, possibly because they were made of perishable material (e.g., wood and leather), as happens today^55^. Falconry might have been carried out on horseback, although the use of bow and arrow should not be overlooked, as some of these objects were recovered at the site^28,56^. Until the half of the last century, the area around Shirenzigou was richer in water and more extensively covered by green forests and grass, making it an ideal habitat for wild animals and, therefore, for hunting^50^.

Faunal resources were exploited for meat, as suggested by the small concentration of cattle bones carrying evidence of disarticulation and defleshing in the food-processing area at the center of the house. Cut and scrape marks were elsewhere remarkably scarce. Basic surface modification analysis on bones from F4 shows a similar lack of butchery marks^37^. It has been demonstrated that it is possible to butcher a whole animal without leaving traces on the bones^57^. This is, indeed, an appreciated skill in Mongol and Kazakh communities in Xinjiang^48,50^. As the fragmentation of the skeletal elements in the waste area HD1 is compatible with breaking for marrow retrieval^50,58^, it is possible that disarticulated fleshed bones were boiled and only after the meat had been eaten, they were broken to retrieve marrow. This practice is well recorded among the modern pastoralist communities in Shirenzigou^48^. Broken bones in the center of the house were found associated with evidence of burning activities^31^ (Fig. 5), but no traces of burning or charring were found on the bones, further supporting the use of low-temperature cooking methods, such as boiling, which do not leave obvious traces on the bones, but only micro structural changes^59^. These can be indentified using appropriate equipment, which was not available for this study. Exploitation of domestic and wild animals for meat is further suggested by stable isotope studies of human remains from Shirenzigou, which have revealed a largely meat-based diet^60^.

Caprines, which dominated the assemblage, were also exploited for meat, although the relatively late killing pattern suggests that they were also used for their secondary products, in particular wool, production of which had apparently been carried out at Shirenzigou since its earliest phases^12^. The discovery of polished bone and stone tools, such as combs and wheels, which may have been used for wool processing, further supports wool production at the site^12,31^. Ethnographic research also indicates that, in order to fully exploit caprines’ resources, modern pastoralists in Shirenzigou delay slaughter to ca. 4 years, before the meat begins to fall off and lose fat^48^. It is noteworthy, however, that zooarchaeological analysis of material from F4, another Iron Age structure in Shirenzigou, has revealed a significantly earlier killing pattern for caprine[^37^; Fig. S1b]. This suggests that, in addition to meat and wool, caprines were potentially used for milk at the site. There is evidence of early dairy consumption in Xinjiang^61,62^, however, for Shirenzigou, further analyses are required to confirm this supposition. The different mortality profiles identified in the two structures may evidence a site-level form of task organization, but comprehensive studies with data from more structures are needed to address this question. On the basis of the available evidence, it appears that caprine exploitation at the site was multipurpose and was not fully optimized for either meat nor secondary products, reflecting a herding model identified in coeval pastoralists sites in Central Asia^22,23,63,64^.

MNE analysis shows a fair representation of all caprine body parts. Although our sample was small (n = 65), comparable BPR patterns were observed in F4 and F3, suggesting the practice of processing these animals on site, with no transport preferences of specific elements^23,37^. By contrast, all cattle elements in F2 came from the right side. While this might reflect some side-preferences or meat-sharing practices for this specific taxa^65,66^, the evidence is too small and the question remains open until more contexts are examined.

Animal resources were used to make tools. In F2 finished products and a small group of unfinished and discarded elements concentrated in the northeastern corner of the house might indicate a domestic production. The variety of artifacts was limited to few simple types, obtained through a basic working process, which required neither specialization, nor complex labor organization. Raw material selection was based on already available resources, including sheep, and to a significantly lesser extent, other large mammals’ elements. Relative taxonomic proportion was in line with previous large-scale research, which included worked bones from Bronze and Iron Age residential and funerary structures at Shirenzigou, but not the data from F2^28^. This suggests a consistent strategy of animal exploitation on a site-level. However, the number of bone artifacts in F2 was comparatively higher (n = 253): the Iron Age structure F4 accounted only for 14 specimens^38^, while in F3 there was no evidence of worked bones^34^. In addition, the vast majority of the artifacts in F2 were related to warfare (i.e., plaques, and possibly arrowheads) and socialization (e.i., astragali), while “tools” were notably scarce. This lack can be due to taphonomic and recovery biases or the result of intentional selection, as ethnographic research found that modern residents of Shirenzigou often use bones to only make toys^48^. Yet, domestic tools were overall scarce in F2, limited to one iron awl and one knife^31^. By contrast, bone tools, such as awls, spatulae and pins, were found in other contexts in Shirenzigou^28^, where bone and metal defensive and offensive weapons were rare^36,38^. The evidence of differentiated use of space in F2—food-processing in the center of the house, the trash heap in the northwestern corner and the manufacturing area in north-eastern corner—indicates that basic tasks were carried out in the strucure, but the distinctive faunal assemblage suggests that F2 was not an ordinary dwelling.

Basing on the northward facing door of the house and the high position within the landscape, it has previously been suggested that F2 could have been a lookout with a defensive function^31^. Several low-investment defensive structures gradually appeared in association with mobile pastoralist communities from the late Bronze Age in Xinjiang^67,68^. This phenomenon has been associated to a growing regional socio-political complexity and explained with the need to regulate the exploitation of, and secure control over rich resources, including pastures^67,68^. Our analysis shows that F2 was sub-optimally placed to prevent access to the meadows within its own gully, but possibly served as watch post for the flatland, which may have formed a safe area for a summer encampment, to prevent attacks by rival groups. The discovery of worked bones related to warfare, such as plaques, but also numerous astragali, which could have been used to negotiate social interactions, further supports the peculiar function of F2. This resonates with an increasing process of socio-economic integration of, but also divisions between, pastoralist groups across the Tianshan region the late first millennium BCE^28,67^.

## Conclusion

The examination of the faunal assemblage from F2 in the Shirenzigou site provides new insights into the Iron Age pastoral societies in the Eastern Tianshan region. Our results show an intensive multipurpose caprine management, while the exploitation of other domestic taxa, cattle, horses and dogs, was limited. This pastoral economy was supplemented with some hunting. The faunal management strategies identified in F2 and its structure reflect models of mobile pastoralism known for the late prehistory of the montaineous regions of Central Asia and Xinjiang, characterized by ephemeral stone structures, high mobility, heavy reliance on caprine herding supplemented by hunting and low-investment agriculture, and horseback riding^18,23,42,64,69^.

While the differentiated use of space in F2 indicates that basic domestic tasks were carried out in the structure, its position within the landscape and the predominance of bone tools related to warfare and socialization activities, suggests that it was not an ordinary dwelling, but it may also have served as a watch post for the summer encampment within the gully. In the context of increased socio-economic complexity across the Eastern Tianshan region in the late first millennium BCE, F2 may represent a localized socio-economic phenomenon, emerged in response to specific cultural and environmental circumstances in Shirenzigou, e.g., those that brought about the necessity to protect the other dwellings.

## Material and methods

This study is based on the faunal remains from the excavations in 2009 of the context F2 in Shirenzigou. All methods comply with relevant institutional, national, and international guidelines and legislation.

### Zooarchaeological analysis

All bones were hand-collected and dry screened, but no floatation was undertaken. The analysis on faunal skeletal elements was conducted in the Zooarchaeology Laboratory of the School of Cultural Heritage at Northwest University. Although there are three distinct occupational phases in F2, given the relatively small scale of the assemblage and the consistency of the taphonomy and taxonomy across all layers, the faunal remains were firstly analyzed as a single assemblage, and then re-examined through spatial analysis. This then permitted a comprehensive image of the animal exploitation strategies within this structure. Presence/absence and, where appropriate, degree of taphonomic processes, including fragmentation, weathering^70^, gnawing^71^, burning^59,72^ and butchery marks^49,58,71^ were recorded in order to assess the conditions of the assemblage and investigate animal use in the house. The degree of fragmentation was recorded as a proportion of the total element (< 0.25, 0.25–0.50, 0.50–0.75, > 0.75) for identified specimens, while for non-identified specimens maximum length was considered (< 5 cm, 5-10 cm, 10-15 cm, > 15 cm). Identification was attempted for every fragment using the reference collection of the Zooarchaeology Laboratory of the School of Cultural Heritage at Northwest University, online reference resources (e.g., archeozoo.org), and published identification manuals^73,74^. All identified bones were used to calculate the number of identified specimens (NISP) and minimum number of individuals (MNI). NISP was calculated using^75^, which allows to count fragments that can be cross-mended as one, so as to limit the risk of counting fragments from the same element more than once and, therefore, to reduce the boost of large-size bones and animals. Although the size and shape of caprine horns has been demonstrated to be an effective method to distinguish between wild and domestic specimens^76^, our assemblage presented a paucity of identifiable horns. Also, the limited availability of reference specimens for caprines in our laboratory, and the uncertainties surrounding the standards to distinguish sheep and goats^77–79^ prevented us from distinguishing the two with a sufficient degree of certainity. For this reason a common category, “caprine”, has been used.

MNI was calculated on the basis of diagnostic zones (DZ) present according to^80^. The same method was employed to calculate the Minimum Number of Elements (MNE), which was corrected by dividing the total MNE by the frequency of the element in the body. Regional MNE was then determined by the maximum corrected MNE value per region using the anatomical region system by^81^, which allows to easily visualize the BPR (Body Part Representation) without the noise of single elements. Also, every region includes at least one robust bone, which reduces possible preservation biases. Given the scarcity of neck and axial elements in our assemblage, the two regions were combined into a single “axis” region.

Mortality profiles for caprine were generated based on bones epiphyseal fusion and tooth eruption and wear. The former was conducted by referring to^82^ system, which relates tooth eruption and wear to long bone fusion to refine age determination accuracy. Caprine mandibles were aged using^83^, which identifies tooth eruption and wear stages for the lower deciduous premolar (dp4), permanent premolar (P4) and molars (M1, M2 and M3).

Worked bones were identified into the lowest identifiable taxonomic unit and classified according to their possible function considering shape, size, use-wear and ethnographic data. The categories used to classify the remains were taken from^28^ not only because they are appropriate to our assemblage, but also to facilitate comparisons with samples from the same area. The categories were: “astragali”, “riding/warfare”, “tools”, “ornaments”, and “unfinished/debris”.

### Spatial analysis

The viewshed analysis was undertaken in ArcGIS using a DEM0.5, created using aerial photography as part of fieldwork undertaken in 2010. The decision to use yes/no rather than fuzzy boundaries is due to the nature of the grassland vegetation in the region. The view point was set to 1.6 m above the ground level at the entrance to F2 since this is the average height of persons during this period^84^. At the same time, the viewshed angle of the viewshed point is set to 270°- 90° (in which 0° points to the north) to ensure the widest field of view. In the output, only the visible part is displayed in orange, and the invisible part is not displayed. The other dwellings and tombs identified as part of the Shirenzigou site were mapped following^9^.

During the excavation in 2009 the contexts of the artefacts were recorded to the nearest cm. The number of faunal remains and ceramic sherds were recorded to the layer or feature in which they were recovered. These features were identified as ash heaps (HD), ash pits (H), fireplaces (SM) and post holes (ZD). These elements were mapped in-field and the plan digitized using CorelDraw X8 https://www.coreldraw.com/en/pages/coreldraw-x8/. This plan was then annotated to show the arrangement of finds within the sites.

### Chronology

In order to contextualize chronologically F2 within the Shirenzigou site and to understand patterns of occupation, six radiocarbon dates, 3 for F2 and 3 for F4, were generated at the Accelerator Mass Spectrometry Dating Laboratory of Peking University and calibrated using OxCal and IntCal20^51,52^.

## Supplementary Information

Supplementary Legends.

Supplementary Figure S1.

Supplementary Tables.

## Data Availability

Data are available in the Supplementary Material.
