# Supplementary material for: New evidence for regional pastoral practice and social complexity in the Eastern Tianshan Mountains in the first millennium BCE
Source: Sci Rep. 2023 Mar 16;13:4338. doi: 10.1038/s41598-023-31489-9 (PMC10020425; doi:10.1038/s41598-023-31489-9)
Supplement: Supplementary file 1 — Supplementary Legends. [file 41598_2023_31489_MOESM1_ESM.docx]

SUPPLEMENTARY MATERIAL: FIGURES CAPTIONS

**Figure S1.** **a,** Relative taxonomic aboundance in F4; **b,** Caprine survivorship from epiphyseal fusion of skeletal elements in F4. The upsticks are presumably due to the small sample size. Both graphs have been generated on the basis of the data in [37]. Dental data for tooth eruption and wear analysis were not available.
