# Supplementary figures and images for: New evidence for regional pastoral practice and social complexity in the Eastern Tianshan Mountains in the first millennium BCE
Source: Sci Rep. 2023 Mar 16;13:4338. doi: 10.1038/s41598-023-31489-9 (PMC10020425; doi:10.1038/s41598-023-31489-9)

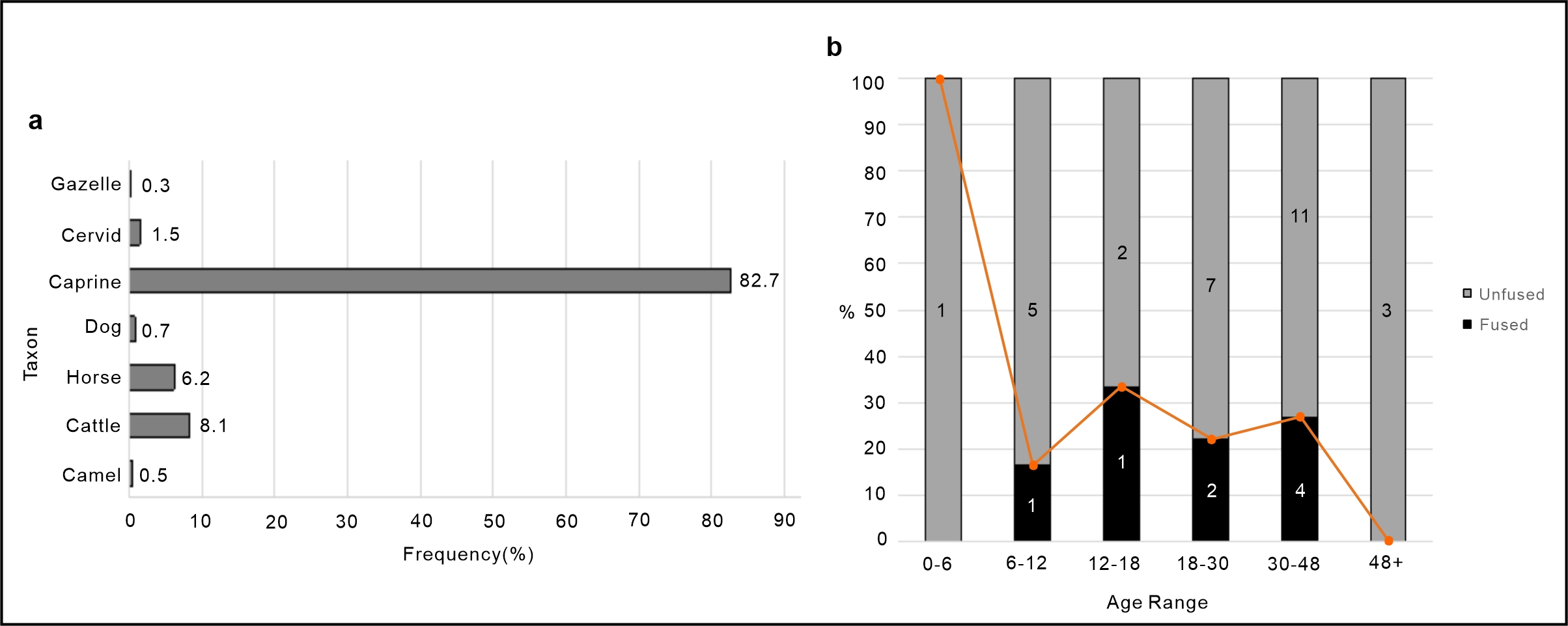

Supplement: Supplementary file 2 — Supplementary Figure S1. [file 41598_2023_31489_MOESM2_ESM.jpg]
