# Supplementary material for: New evidence for regional pastoral practice and social complexity in the Eastern Tianshan Mountains in the first millennium BCE
Source: Sci Rep. 2023 Mar 16;13:4338. doi: 10.1038/s41598-023-31489-9 (PMC10020425; doi:10.1038/s41598-023-31489-9)
Supplement: Supplementary file 3 — Supplementary Tables. [file 41598_2023_31489_MOESM3_ESM.docx]

**Table S1. Quantification of the taphonomic processes affecting the faunal remains in F2, Shirenzhigou. The table shows the degree of fragmentation for Identified and Non-identified elements, and the absence/presence of all the other taphonomic processes.**

| **Taphonomic process** | **Degree** | **Number** | **%** |
| --- | --- | --- | --- |
| Fragmentation  (Identified elements) | >0.25 | 253 | 32.5 |
|  | 0.25%-0.50 | 36 | 4.6 |
|  | 0.50%-0.75 | 20 | 2.5 |
|  | >75 | 53 | 6.8 |
| Fragmentation  (Non-identified elements) | >5cm | 234 | 30.1 |
|  | 5-10cm | 128 | 16.4 |
|  | 10-15cm | 32 | 4.1 |
|  | >15cm | 23 | 3 |
| **Total** | | **779** | **100** |
| Weathering | N/A | 9 | 1.2 |
| Gnawing | N/A | 15 | 1.9 |
| Burning | N/A | 0 | 0 |
| Butchery | N/A | 10 | 1.3 |

**Table S2. Quantification of different types of worked bones per taxon in F2.**

| **Taxon** | **Astragali** | | **Riding/warfare** | | | | **Tools** | | **Ornaments** | | **Unfinished/ Debris** | | **Total** | |
| --- | --- | --- | --- | --- | --- | --- | --- | --- | --- | --- | --- | --- | --- | --- |
|  |  |  | **Plaques** | | **Bits** | |  |  |  |  |  |  |  |  |
|  | **N** | **%** | **N** | **%** | **N** | **%** | **N** | **%** | **N** | **%** | **N** | **%** | **N** | **%** |
| Caprine | 166 | 65.6 |  |  | 1 | 0.4 | 1 | 0.4 | 1 | 0.4 |  |  | **169** | **66.8** |
| Cattle | 3 | 1.2 |  |  |  |  |  |  |  |  |  |  | **3** | **1.2** |
| Deer |  |  |  |  |  |  |  |  |  |  | 2 | 0.8 | **2** | **0.8** |
| Large Mammal |  |  | 72 | 28.5 |  |  |  |  |  |  | 7 | 2.7 | **79** | **31.2** |
| **Total** | **169** | **66.8** | **72** | **28.5** | **1** | **0.4** | **1** | **0.4** | **1** | **0.4** | **9** | **3.5** | **253** | **100** |

**Table S3. Quantification and distribution of different types worked bones in various locations and features in F2.**

| **Location** | **Astragali** | | **Riding/warfare** | | | | **Tools** | | **Ornaments** | | **Unfinished/ Debris** | | **Total** | |
| --- | --- | --- | --- | --- | --- | --- | --- | --- | --- | --- | --- | --- | --- | --- |
|  |  |  | **Plaques** | | **Bits** | |  |  |  |  |  |  |  |  |
|  | **N** | **%** | **N** | **%** | **N** | **%** | **N** | **%** | **N** | **%** | **N** | **%** | **N** | **%** |
| F2① | 0 | 0 | 1 | 0.4 | 0 | 0 | 1 | 0.4 | 0 | 0 | 0 | 0 | **2** | **0.8** |
| F2④ | 7 | 2.8 | 0 | 0 | 0 | 0 | 0 | 0 | 0 | 0 | 0 | 0 | **7** | **2.8** |
| DM21 | 7 | 2.8 | 68 | 26.8 | 0 | 0 | 0 | 0 | 1 | 0.4 | 1 | 0.4 | **77** | **30.4** |
| DM22 | 7 | 2.8 | 0 | 0 | 1 | 0.4 | 0 | 0 | 0 | 0 | 8 | 3.2 | **16** | **6.3** |
| HD1 | 109 | 43.1 | 3 | 1.2 | 0 | 0 | 0 | 0 | 0 | 0 | 0 | 0 | **112** | **44.3** |
| HD3 | 39 | 15.3 | 0 | 0 | 0 | 0 | 0 | 0 | 0 | 0 | 0 | 0 | **39** | **15.4** |
| H1 | 0 | 0 | 0 | 0 | 0 | 0 | 0 | 0 | 0 | 0 | 0 | 0 | **0** | **0** |
| **Total** | **169** | **66.8** | **72** | **28.4** | **1** | **0.4** | **1** | **0.4** | **1** | **0.4** | **9** | **3.6** | **253** | **100** |
